# Texture feature extraction from microscope images enables a robust estimation of ER body phenotype in Arabidopsis

**DOI:** 10.1186/s13007-021-00810-w

**Published:** 2021-10-26

**Authors:** Arpan Kumar Basak, Mohamadreza Mirzaei, Kazimierz Strzałka, Kenji Yamada

**Affiliations:** 1 Faculty of Biology, Jagiellonian University, Krakow, Poland; 2 Malopolska Centre of Biotechnology, Jagiellonian University, Krakow, Poland; 3 Faculty of Biochemistry, Biophysics and Biotechnology, Department of Plant Physiology and Biochemistry, Jagiellonian University, Krakow, Poland

**Keywords:** ER-Body, Confocal microscopy, Morphology, Haralick feature, Quantitative analysis

## Abstract

**Background:**

Cellular components are controlled by genetic and physiological factors that define their shape and size. However, quantitively capturing the morphological characteristics and movement of cellular organelles from micrograph images is challenging, because the analysis deals with complexities of images that frequently lead to inaccuracy in the estimation of the features. Here we show a unique quantitative method to overcome biases and inaccuracy of biological samples from confocal micrographs.

**Results:**

We generated 2D images of cell walls and spindle-shaped cellular organelles, namely ER bodies, with a maximum contrast projection of 3D confocal fluorescent microscope images. The projected images were further processed and segmented by adaptive thresholding of the fluorescent levels in the cell walls. Micrographs are composed of pixels, which have information on position and intensity. From the pixel information we calculated three types of features (spatial, intensity and Haralick) in ER bodies corresponding to segmented cells. The spatial features include basic information on shape, e.g., surface area and perimeter. The intensity features include information on mean, standard deviation and quantile of fluorescence intensities within an ER body. Haralick features describe the texture features, which can be calculated mathematically from the interrelationship between the pixel information. Together these parameters were subjected to multivariate analysis to estimate the morphological diversity. Additionally, we calculated the displacement of the ER bodies using the positional information in time-lapse images. We captured similar morphological diversity and movement within ER body phenotypes in several microscopy experiments performed in different settings and scanned under different objectives. We then described differences in morphology and movement of ER bodies between *A. thaliana* wild type and mutants deficient in ER body-related genes.

**Conclusions:**

The findings unexpectedly revealed multiple genetic factors that are involved in the shape and size of ER bodies in *A. thaliana*. This is the first report showing morphological characteristics in addition to the movement of cellular components and it quantitatively summarises plant phenotypic differences even in plants that show similar cellular components. The estimation of morphological diversity was independent of the cell staining method and the objective lens used in the microscopy. Hence, our study enables a robust estimation of plant phenotypes by recognizing small differences in complex cell organelle shapes and their movement, which is beneficial in a comprehensive analysis of the molecular mechanism for cell organelle formation that is independent of technical variations.

**Supplementary Information:**

The online version contains supplementary material available at 10.1186/s13007-021-00810-w.

## Background

The texture features can provide an outline of the morphology by considering the correlation between neighbouring pixels. The extraction of the texture features enables capturing morphological differences in images. Micrograph-based image profiling frequently uses Haralick features as texture features to understand morphological differences. Haralick features compute on the grey-level co-occurrence matrix (GLCM), where each element in the matrix is considered to be the probability that a pixel value is found adjacent to its neighbouring pixel [1]. A series of statistical properties are then computed from the GLCM that provide information on morphology. Together with spatial and intensity features, a Haralick profile provides 14 diverse features that estimate the morphological variation within objects. In the field of medical research, image data from positron emission tomography (PET) and magnetic resonance imaging (MRI) are used for profiling with this feature to detect anomalies [2, 3]. This feature set is exploited and considered important in the diagnosis of tumour cells. These medical studies suggest that Haralick features could also be useful in the quantitative analysis of plant cell imaging.

Organelle movement is a measurable phenotype in addition to morphology and is usually quantified from time-lapse images. Some studies have shown the linear and non-linear dynamics of organelle movement [4], classification of organelle trajectories [4], and temporal dynamics of overall ER networks [5]. However, integrating the z-stack in time-lapse images of the confocal microscope brings complexity to the measurement of movement, because the organelle size and speed will change for each scan along with the depth and time [6].

Brassicaceae and its closely related family plants have a specific structure derived from the endoplasmic reticulum (ER), namely, the ER body (also known as the fusiform body or dilated cisternae), which can be visualized by green fluorescent protein (GFP) with an ER-retention signal (GFP-HDEL) [7–9]. ER bodies are involved in the plant resistance against insect herbivory or pathogens by accumulating β-glucosidases (BGLUs) that activate defensive metabolites [10–13]. ER bodies are spindle-shaped structures of 5 to 10 µm in the longitudinal, and they are morphologically distinct from the ER and other cellular vesicles [14].

Two ER body morphology mutants, namely *nai1-1* and *long ER body-1* (*leb-1*), have been isolated in *Arabidopsis thaliana*. The *nai1-1* mutant does not accumulate ER bodies in seedlings, and a mutation has been found in a gene encoding a basic helix-loop-helix type transcription factor, namely bHLH020/NAI1 [15]. NAI1 regulates the induction of *BGLU23/PYK10* and *BGLU21,* the gene products of which specifically accumulate in ER bodies [9, 14]. ER bodies in *leb-1* mutants are fewer than in the wild type, but they are more elongated [14]. A mutation has been found in the *BGLU23*/*PYK10* gene in the *leb-1* mutants [14]. Consequently, it produces a mutated protein with Cys to Tyr exchange at the 29th position, which reduces the protein stability and proper oligomerization of BGLU23/PYK10 [14]. The single knockout mutants of *pyk10-1* and *bglu21-1* show modest changes in the morphology of ER bodies compared to the wild type, but the *pyk10-1 bglu21-1* double knockout mutant shows an elongated ER body phenotype similar to *leb-1* [14]. The quantitative ER body phenotypes of *leb-1 bglu21-1* are similar to those of *leb-1* [14]. These findings suggest that the packing of the BGLU23/PYK10 protein brings a morphological variation into the ER bodies.

Besides BGLU23/PYK10 and BGLU21, ER bodies in *A. thaliana* accumulate specific membrane proteins, namely MEMBRANE PROTEIN OF ER BODY 1 (MEB1) and MEB2. These proteins have a homology to the VACUOLAR IRON TRANSPORTER 1 (VIT1) family of proteins that are involved in metal transportation in plants [16, 17]. MEB1 and MEB2 seem to have a transport activity with iron and manganese ions because overexpression of *MEB1* or *MEB2* in yeast (*Saccharomyces cerevisiae*) enhances resistances against these metals [17]. However, their role in the ER body formation or ER body-mediated plant defence is still obscure.

Confocal microscope imaging is a powerful tool to show the morphological differences of intracellular structures between samples. However, it is still challenging to capturing the morphological parameters quantitatively. Strikingly, very few analyses have been undertaken to show morphological variations of ER bodies in *A. thaliana.* Quantitative analysis of ER body morphology in the *leb-1* mutant has successfully revealed that the mutation in *BGLU23*/*PYK10* distorts ER body size and number [14]. However, this estimation of morphological parameters was restricted to spatial and intensity measures without considering the texture features of the pixels.

Recent advances in image processing have considered extensive use of artificial intelligence, such as deep learning and machine learning methods, to segment cellular features and classify objects from microscope images in the field of cellular biology [18–20]. However, a diverse dataset is required to make such a classifier that could potentially be able to distinguish ER bodies from other ER derived vesicles within plant cells. In GFPh mutants ER bodies are protein dense objects and are distinct from *nai1-1* mutants. A neural network pre-trained on U-net, or convolutional neural network (CNN) models may not fulfil the classification task, as the trained images are diverse and ER bodies are more or less similar to other ER derived vesicles in shape. Therefore, an ER body classifier based on a vast array of image datasets would be needed to be able to distinguish the ER body signal from ER and to avoid the overfitting issues in deep learning methods. To detect the changes within ER body morphology a wide range of ER body mutant screening is required.

Here, we have presented a robust approach to estimate the morphological features obtained from the segmented cells that distinguish ER bodies from the background. We employed the maximum contrast projection [21] and an *EBImage* program package [22] to segment the cells with ER bodies from the background based on adaptive thresholding and Voronoi tessellation. We used this method to segment cells from micrographs and then quantified the morphological parameters. The methodology uses morphological features of micrographs to represent their properties for samples [23]. This is an unbiased estimation of cellular features and their morphological variants considering spatial, intensity and Haralick features. We introduced this method in plant sciences for the first time to denoise the variants of ER bodies across micrograph images. In addition to morphology, we also quantified the movement of the ER bodies from the positional information of the cell component. Consequently, we observed the overall diversity of the ER body morphology and movement in not only *nai1-1* and *leb-1 bglu21-1*, but also in *meb1-1*, *meb2-1* single and *meb1-1 meb2-1* double mutants.

## Methods

### Plant materials

**Fig. 1:**
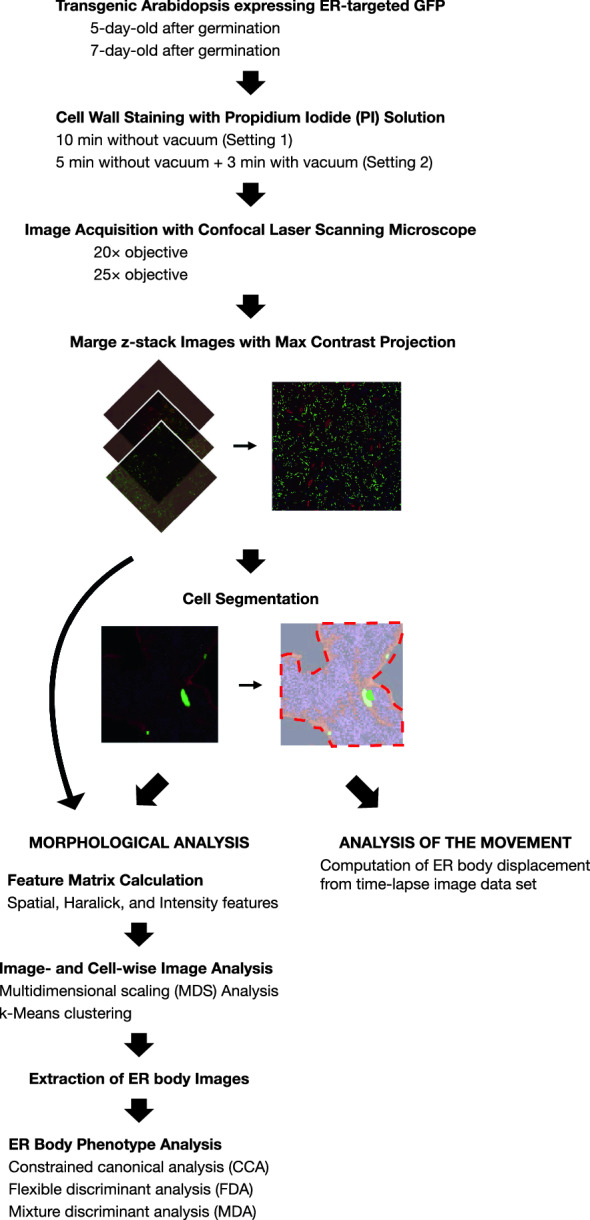
Workflow of the analysis

**Fig. 2:**
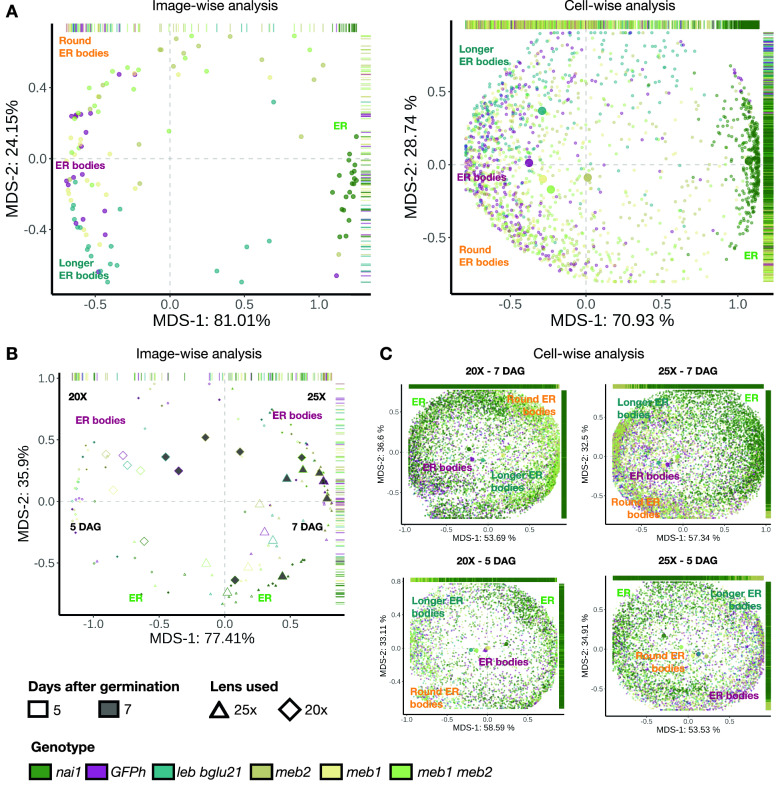
Multivariate analysis revealed morphological diversity of ER bodies between *A. thaliana* wild type and mutants. **A** The image-wise (left) and segmented cell wise (right) MDS analysis of 42 features extracted from microscope images with the setting 1. **B** The image-wise MDS analysis with the setting 2. **C** The segmented cell-wise MDS analysis with the setting 2

Transgenic seedlings of *Arabidopsis thaliana* (Columbia accession) wild type (GFP-h), *nai1-1*, *leb-1 bglu21-1*, *meb1-1*, *meb2-1*, and *meb1-1 meb2-1* mutant plants expressing ER targeted GFP in the cotyledons were used (Table 1) [14, 15, 17]. The *A. thaliana* seeds were sterilized and cultivated in solid media (1/2 × Murasige-Skoog salt, 250 mM MES-KOH pH 5.5, 1% (w/v) sucrose, and 0.4% (w/v) gellan gum (Fujifilm, Japan)) for 5 and 7 days. After the cultivation, cotyledons were dissected and the cell walls were stained with a 100 µg/ml propidium iodide (PI) solution by implementing some modification in the protocol [24] and then were subjected to confocal imaging under 20 × and 25 × objectives. Three independent experiments were performed using two different methods of staining: (1) treating the cotyledons in the PI solution for 10 min and then immediately observing them (Setting 1, Fig. 1); (2) cotyledons were treated in the PI solution for 5 min, then infiltrated with a vacuum pump for 1 min followed by washing with deionized water for 2 min (Setting 2, Fig. 1). Accordingly, three image datasets were generated; setting 1 with a 20 × objective, setting 2 with a 20 × objective and with a 25 × objective.

**Table 1:** Transgenic *Arabidopsis thaliana* lines used in the study

Genotype	ER body phenotype in cotyledons	Description
wild type (GFP-h)	Normal	Wild type
*nai1-1*	No ER bodies [15]	Mutation in a transcription factor
*leb-1 bglu21-1*	Long and few [14]	Mutation in ER body components
*meb1-1*	Resembles wild type but smaller (This study)	Mutation in an ER body membrane protein
*meb2-1*	Round, aggregate, and less movement (This study)	Mutation in an ER body membrane protein
*meb1-1 meb2-1*	Round, aggregate, and less movement (This study)	Mutation in ER body membrane proteins

### Image acquisition

Image acquisition was conducted with a confocal laser scanning microscope (Zeiss LSM 880) under 20 × and 25 × objective lenses with the range of gain between 450 to 500 and the digital gain as 1, to reduce pixel intensity saturation. Glycerol was used on the coverslip during slide preparation for the 25 × objective lens. An Argon 488 laser and a HeNe laser were used for image acquisition and the magnitude of intensity was kept to 10 to reduce photobleaching and autofluorescence. The pinhole was set to be 10 µm for optimum laser accommodation on the objective. Images were acquired in two distinct settings at the 1024 by 1024-pixel range by averaging 2 pixels in such a way that each pixel explains 0.42 µm^2^ of the area of the object in the 20 × objective and 0.54 µm^2^ in the 25 × objective. Scanning was performed bi-directionally across the stage and with a colour depth of 8 bits, at a scan speed of 5. Z-stack acquisition was performed at a depth of 2 µm/slices to have volumetric image information. Images were acquired from the surface of the epidermal cells of the cotyledons in a randomised order.

### Integration of z-stack images and segmentation

Raw images were pre-processed and segregated by correcting dimensions and RGB channels, merged by the *MaxContrastProjection* package in R, and finally the pixels were normalised. Image pre-processing and statistics were conducted using R packages, *EBImage* [22], *vegan* [25] and *r-base* libraries, respectively. The z-stack images were merged using specific criteria for the *MaxContrastProjection* package (https://github.com/arpankbasak/ERB_DynaMo). The red channel was specified for the cell walls and the green channel was specified for the ER bodies to segregate the merged image of 3D plant tissue in 2D representation. Segregation was done to maintain homogeneity for the analysis among the images for downstream analysis (*segregate* script; https://github.com/arpankbasak/ERB_DynaMo). Specific masks were generated to assign cell and ER body border lines for the corresponding channels. Knitted images from the projection were taken as input for the analysis pipeline and feature extraction was conducted using adaptive thresholding and segmentation principles (parameters in Additional file 1). Adaptive thresholding on the pixel intensity was used to detect the cell and ER body border lines. Quartile based selection was considered for ER body segmentation and cell segmentation (parameters in Additional file 1). An ER signal emits ≥ 95% of the total GFP in a tissue section; in an image, an ER body showed a dense signal with a small pixel area. The PI fluorescence showed a jigsaw puzzle pattern of the cell walls. Two masks were set for ER bodies and cell walls separately with a given range of parameters (Additional file 1). Segmentation was conducted using Voronoi tessellation using both masks. The accuracy of segmentation was determined by manually counting the number of segmented cells in some examples. The segmented cells with a ≥ 5000 pixel unit surface area were further chosen for the feature analysis of ER bodies. The image-, segmented cell- and ER body-wise morphological features were computed. A detailed schematics of the image pipeline is represented in Additional file 2 and the detailed workflow of image segmentation is represented in Additional file 3.

### Analysis of the dynamics of the cellular features

Time-lapse images were obtained with a confocal microscope using the same procedure as mentioned above. The z-stack merged images were used from an independent experiment and set the blue channel as an ER body’s initial positions (ER body at time 0). Every time point images stores information of the initial position of the ER bodies in the blue channel. Subsequently, these images were segmented into cell-wise images and ER bodies were extracted at each specific time point, while retaining their initial position. The initial and specific time point images were projected to show the movement across the time point. These images were used to extract position features that showed dislocation (*segregate_dynamics* and *segmentation_dynamics* scripts; https://github.com/arpankbasak/ERB_DynaMo). The processed images were converted into a movie for visualisation of the dynamics (*MomentProjection* script; https://github.com/arpankbasak/ERB_DynaMo). The initial and the final position of the ER body features from the location parameter (m.cx and m.cy in the feature matrix, Additional file 4) were used to calculate the cosine distances, representing the displacement of the cellular feature along the pathway. The moving average was calculated to obtain a better approximation for the organelle dynamics across time. A non-linear regression method, locally estimated scatterplot smoothing (LOESS), and a generalized linear model (GLM) were used for statistical analysis.

### Feature extraction in the segmented cells

Data analyses of images was conducted in R environment using customized scripts in an Argon server x86_64-conda_cos6-Linux-gnu (64-bit): CentOS Linux 7 (Core). The features were detected based on Otsu’s method [26, 27] and adaptive thresholding at the 97% quantile of the pixel intensities. After adaptive thresholding and segmentation of cells and ER bodies, a feature matrix was generated from the stack of cells and ER body like features describing intensity, spatial, Zernike moment [28], and Haralick features (Additional file 4). Statistical analysis was performed on the feature matrix of spatial, intensity and Haralick profile of the features within the segmented cells of the images. The obtained features were distinguished by unique Feature-IDs that can be referred to as the feature matrix for further analysis (*segmentation* script; https://github.com/arpankbasak/ERB_DynaMo). The z-scores of the morphological parameters in the feature matrix were grouped and aggregated for the corresponding samples and their mean z-score values were calculated. The feature matrix was aggregated for analysing the sample images, segmented cells and features. Further stratification was conducted by experimental settings, staining method, genotype, days after germination, and objective lens. Constrained canonical analysis (CCA) was used to show the variation of ER body features explained by the genotype. A PERMANOVA test over 1000 iterations was used to compute the statistical significance. The feature matrix was further merged, and selected features were used to compute the morphological variation [1] (*featurematrix* script; https://github.com/arpankbasak/ERB_DynaMo). The feature matrix was used to compute descriptive statistics and compare the genotypes (*image* and *segmentedcells* scripts; https://github.com/arpankbasak/ERB_DynaMo). The segmented cells were clustered and copied into a new directory for visualisation (*pool_features* script; https://github.com/arpankbasak/ERB_DynaMo). The morphological differences in the cellular features within the clustered cells were analysed (*clustering_features* script; https://github.com/arpankbasak/ERB_DynaMo). The suggested statistical analyses performed on the feature matrix is described below. The feature matrix can also be used for customised data analysis.

### Multivariate analysis

Multidimensional scaling (MDS) analysis was performed with normalized feature matrixes between images of samples. Pearson correlation was used to compute the dissimilarity. This type of analysis was performed between segmented cells or between plants. Further, k-means clustering was performed on a normalised feature matrix of the segmented cells. The genotype was set as a fixed factor, while the other experimental settings were set as random factors to compute statistical relevance. Descriptive statistics were performed on the set of 40 features measured across the samples followed by multiple hypothesis correction using a false discovery rate (FDR) cut-off ≤ 0.05.

### Flexible- and mixture-discriminant analysis

The proportion of features in the segmented cells that showed distinct ER body morphology was computed by flexible (FDA) and mixture discriminant analysis (MDA). The z-score normalised features were further used to model the proportion of ER bodies that were distinct for the observed genotypes. MDA and FDA were conducted assuming that the features of the corresponding genotypes are either linearly separable or not [29, 30].

## Results

### Image projection and single-cell segmentation

The analysis was conducted on a total of 240 images from the wild type and mutants, with 18 z-stacks on average. These images were merged with maximum contrast projection, resulting in 41 images in the wild type, 42 images in the *nai1-1* mutants, 40 images in the *leb-1 bglu21-1* mutants, 40 images in the *meb1-1* mutants, 38 images in the *meb2-1* mutants, and 39 images in the *meb1-1 meb2-1* mutants (Table 2). Subsequently, cell segmentation based on the red fluorescence of cell walls provided 12,408 cell images in the wild type, 17,205 cell images in the *nai1-1* mutants, 9109 cell images in the *leb-1 bglu21-1* mutants, 10,664 cell images in the *meb1-1* mutants, 6862 cell images in the *meb2-1* mutants and 10,357 cell images in the *meb1-1 meb2-1* mutants (Table 2). Further, segregation of the 66,605 cells from the 240 images resulted in 29,629 cells that had ER body like features (Table 3).

**Table 2:** Summary of cell segmentation

Genotype	Setting	Objective	Days after germination	Segmented cells	Images
wild type (GFP-h)	1	20 ×	7	6181	19
			5	830	5
			7	1712	6
	2	25 ×	5	1401	5
			7	2284	6
*nai1-1*	1	20 ×	7	8901	20
			5	1215	5
			7	2712	6
	2	25 ×	5	1514	5
			7	2863	6
*leb-1 bglu21-1*	1	20 ×	7	5188	20
			5	630	5
			7	804	5
	2	25 ×	5	1078	5
			7	1409	5
*meb1-1*	1	20 ×	7	6723	20
			5	292	5
			7	1208	5
	2	25 ×	5	695	5
			7	1746	5
*meb2-1*	1	20 ×	7	2552	18
			5	613	5
			7	1428	5
	2	25 ×	5	829	5
			7	1440	5
*meb1-1 meb2-1*	1	20 ×	7	4893	19
			5	1021	6
			7	1797	5
	2	25 ×	5	1117	5
			7	1529	4

**Table 3:** Summary of ER body like features

Genotype	Objective	Days after germination	Cells with ER-body like features	No. of images
wild type (GFPh)	20 ×	5	271	3
		7	5949	21
	25 ×	5	813	4
		7	811	6
*leb-1 bglu21-1*	20 ×	5	251	3
		7	4920	23
	25 ×	5	504	5
		7	732	4
*meb1-1*	20 ×	5	52	2
		7	5234	22
	25 ×	5	275	5
		7	767	5
*meb2-1*	20 ×	5	71	2
		7	1989	17
	25 ×	5	529	4
		7	494	5
*meb1-1 meb2-1*	20 ×	5	518	4
		7	4509	19
	25 ×	5	406	5
		7	531	3

### Image-wise and segmented cell-wise analysis

The z-scores of 40 features (6 spatial, 8 intensity and 26 Haralick features) were calculated from the merged micrograph images from the wild type and mutants based on GFP fluorescence of ER and ER bodies (Additional file 5A). Based on these features, we calculated the Pearson correlation coefficient (PCC) and conducted MDS analysis (Fig. 2 and Additional file 5B to H).

In the heatmap of the feature matrix, significant differences were found in the patterns between the plants that had ER bodies (e.g. wild type) and the plants that did not have ER bodies (*nai1-1*) in a specific experiment, which was the dataset with 7-day-old plants with PI staining setting 1 and 20 × objective lens (Additional file 5A), indicating that the images can be separated into two groups depending on the presence or absence of ER bodies in the dataset.

The MDS analysis showed that micrograph images can be separated on the scatter plot according to the morphology of ER bodies. The MDS1 and MDS2 axes explained 81.01% and 24.15% in the image-wise analysis, respectively (Fig. 2A). The separation between the images from the wild type and the *nai1-1* mutant occurred along the MDS1 axis, showing that the axis indicates the presence or absence of ER bodies (Fig. 2A). The separation between the wild type plants and *leb-1 bglu21-1* double mutants occurred along the MDS2 axis, suggesting that the axis explains the length of ER bodies since the *leb-1 bglu21-1* double mutants have longer ER bodies compare to the wild type. We found that *meb1-1* and *meb1-1 meb2-1* mutants showed separations in MDS2, suggesting that these mutants have shorter ER bodies (Fig. 2A). The micrograph images are even further separated in the scatter plot and explain 24.15% in the MDS2 axis and 19.79% in the MDS3 axis (Additional file 5B). We further conducted MDS analysis with two other image data sets (setting 2 with 20 × objective, and setting 2 with 25 × objective) from a different batch of experiments (Fig. 2B). In this data the variation in the image captured the difference of the objective lens and the age of the cotyledons in the MDS1 axis (77.41%). Further, we found the image variation with the presence or absence of ER bodies in the MDS2 axis (35.9%).

A similar trend was observed when the MDS analysis was done on segmented cell images of a dataset with 7-day-old plants, PI staining setting 1 and 20 × objective lens. The cells that had ER bodies were clustered separately from the cells devoid of ER bodies in the MDS plots and showed that the maximum variation of MDS1, MDS2 and MDS3 were 70.93%, 28.74% and 21.36%, respectively (Fig. 2A and Additional file 5C). This suggests that the morphological parameters for the ER bodies are specific and discrete from that of the ER network. When we conducted the MDS analysis of segmented cells with the other image data sets (setting 2 with 20 × objective and setting 2 with 25 × objective) for the respective groups of objective lens and age of the seedling, the separation between the cells with and without ER bodies were moderate. In the images with 20 × objective, the variations explained were 53.69% and 58.59% in MDS1, 36.6% and 33.11% in MDS2, and 14.05% and 13.68% in MDS3 (Fig. 2C, Additional file 5E and H). The variations explained within the images from the 25 × objective were 57.34% and 53.53% in MDS1, 32.5% and 34.91% in MDS2, and 15.17% and 16.81% in MDS3 (Fig. 2C, Additional file 5F and G). Therefore, although the estimation is robust according to image taking methodology, the same experimental setting is desirable to predict the MDS analysis precisely.

**Fig. 3:**
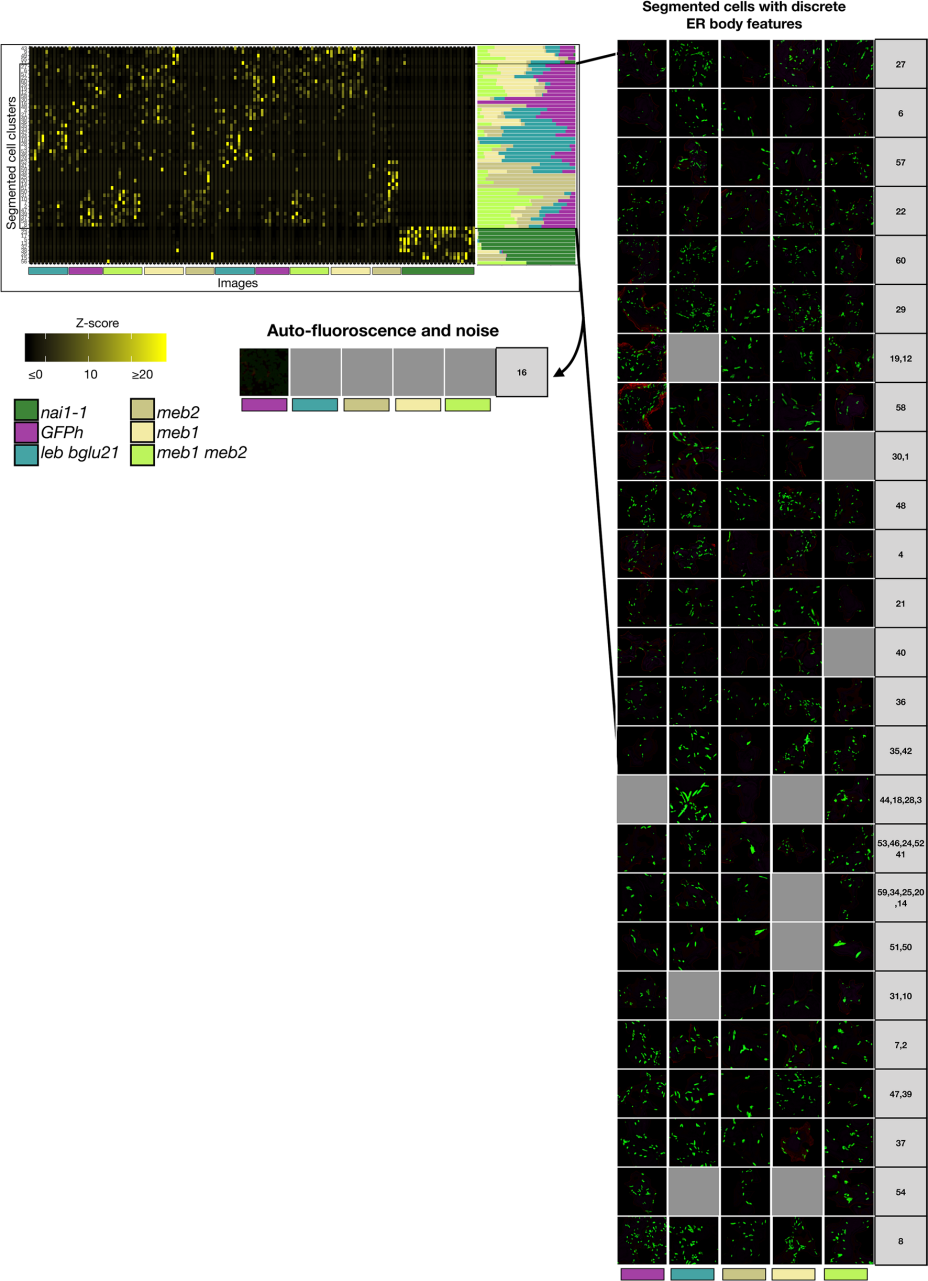
Cluster analysis revealed groups of segmented cell images. The heatmap on the left shows the mean z-score. The bar-plot represents the proportion of segmented cells belonging to corresponding genotypes. The microscope images on the right show randomly picked segmented cells belonging to corresponding clusters or consensus cluster. The images below 10% of cluster representatives are not shown

The feature data of cell-wise images were subjected to k-means clustering (k = 60, assuming at least 10 clusters per genotype) within each group (Fig. 3) to determine the group of cells having distinct ER body phenotypes. The optimum k-value was determined by Akaike information criterion (AIC) over a range of k-values (minimum 6 and maximum 100, an example is presented in Additional file 6). The optimum k-value was chosen within the range of 55 to 65, beyond this range the variations between the clusters were less than 0.95%. The k-means clusters segregated the cells that were devoid of ER bodies and were similar to the cells of the *nai1-1* mutant, and the remaining cells can be attributed to their genotype in the features. Clusters that showed ER body like features were considered for further analysis to evaluate the overall effect of the genotype in explaining the morphological diversity of ER bodies. The features from the clusters of segmented cells across different experimental settings were integrated. Further, we investigated individual images of the clusters including ER body images (Fig. 3). The images of the clusters showed similar ER body morphology within each cluster, but apparent variations between the clusters. This indicates that the k-means cluster analysis grouped the cells having similar phenotypic variants throughout the genotypes. In clusters 10, 12, 19, 31 and 54, we observed cells mostly belonging to plants without *leb-1 bglu21-1*. In clusters 2, 18, 28, and 44, we observed cells mostly belonging to mutants. Clusters 2, 7, 50, 51 and 54 revealed morphologically distinct ER bodies. Cluster 16 was identified as an autofluorescence like feature, presumably noise images. With this approach we excluded the cell images from the *nai1-1* mutant and from stomata cells with no ER bodies as well as autofluorescence. Consequently, 29,629 cells were classified from among 66,605 cell-wise images as having ER bodies after k-means clustering analysis. At this resolution the differences in the ER body morphology across the mutants and the wild type could be compared. Further, anomalies in the texture features within ER bodies were detected from the clustered cells.

### Feature analysis of cells having ER bodies

**Fig. 4:**
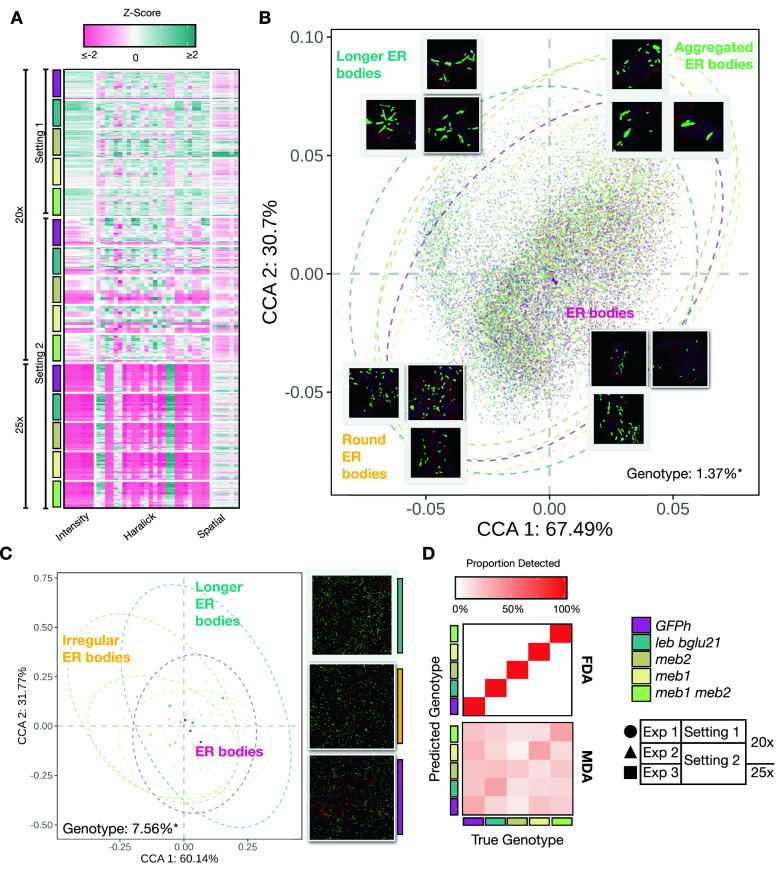
Integrated analysis on the datasets reveals distinct morphological diversity of ER bodies between *A. thaliana* wild type and mutants. **A** The heatmap represents the z-score measure of the 40 morphological parameters (x-axis) and segmented cells clustered to show ER body phenotype. These clustered cells belong to their independent experiments taking in 2 different settings (Setting 1 and Setting 2) and 2 objectives (20× and 25×). **B** Constrained ordination analysis (CCA) was performed on the z-scores of the morphological parameters of the clustered cells for each of the ER bodies detected from the independent experiments, using genotype as a fixed factor. The difference in the colour of the dotted circle represents the genotypic differences. The variance explained by genotype is 1.37%. **C** CCA was performed on aggregated morphological parameters of the clustered cells. The variance explained by genotype is above 7%. Note that the dispersions between the *leb-1 bglu21-1* (blue-dotted circle), *meb2-1* (dark yellow-dotted circle) and *meb1-1 meb2-1* (light green-dotted circle) are distinct from the wild type (purple-dotted circle) in the scatterplot. **D** The heatmap represents a confusion matrix from discriminant analysis performed by using the FDA and MDA methods

We re-examined the morphological variations of ER bodies with pooled cell-wise images that show ER bodies. However, we found a technical variation between the 20 × and the 25 × objective lens on integrating the z-score values of intensity, Haralick and spatial features (Fig. 4A). Therefore, we performed constrained ordination on the ER body phenotype within these cells by using PCC distances to find the variances among the features (Fig. 4B). We set genotype as a fixed factor and the others (objective lens, plant age, staining method) as random factors in the analysis. The variation explained in CCA1 and CCA2 was 67.49% and 30.7%, respectively. After conditioning the random factors we observed a significant difference in the feature diversity within the genotypes. The significance was determined by conducting a permutational multivariate analysis of variance (PERMANOVA) test on constrained ordination for Pearson correlations within each cell and feature (*p*-value < 0.05, 1000 iterations) (Additional file 7). Despite the moderate morphological variation, constrained ordination revealed that genotype difference could be explained at 1.37%. We found that images could be grouped depending on the ER morphology, such as long, rounded and aggregated ER bodies (Fig. 4B). Thus, we grouped the images according to the k-mean clustering (Fig. 3) within genotypes and performed constrained ordination on the mean of features from the grouped images. In the pooled dataset, we found that the features of the mutants could be distinguished from each other according to the difference in their ER body morphology (Fig. 4C). The variation explained by genotype was 7.56% (*p*-value < 0.01) and the variation shown in CCA1 and CCA2 was 60.14% and 31.77%, respectively (Fig. 4C). The scatter plot shows the wild type and the *meb1-1* mutant placed in the centre, while the *leb-1 bglu21-1* with long ER bodies shifted to the upper-left, and the *meb2-1* and *meb1-1 meb2-1* mutants with rounded and aggregated ER bodies shifted to the right (Fig. 4C and Additional file 5I).

The FDA and MDA provided the proportion of cell images predicted to be of a certain genotype. In MDA analyses we observed that a small proportion (< 30%) of cells from mutants were predicted to be from the wild type, suggesting that the mutant plants have cells that show similar ER body features to those of the wild type (Fig. 4D). However, substantial levels (> 60%) of mutant cell features were predicted to be in their respective genotypes, suggesting that each mutant had ER bodies with specific morphological features (Fig. 4D). Also, a proportion of cells from the mutants were still predicted as belonging to different genotypes, indicating the tendency of similarity in features across the genotypes (< 10%). The FDA analysis showed the proportion of ER bodies that were predicted to be discrete with 100% identity to their respective genotype. This suggests that the variation within the genotype may be non-linear. The variation in the features estimated within the genotype is represented in the box plots (Additional file 8), which shows that the mutants had an ER body morphology distinct from the wild type.

### Dynamics of the cellular feature

**Fig. 5:**
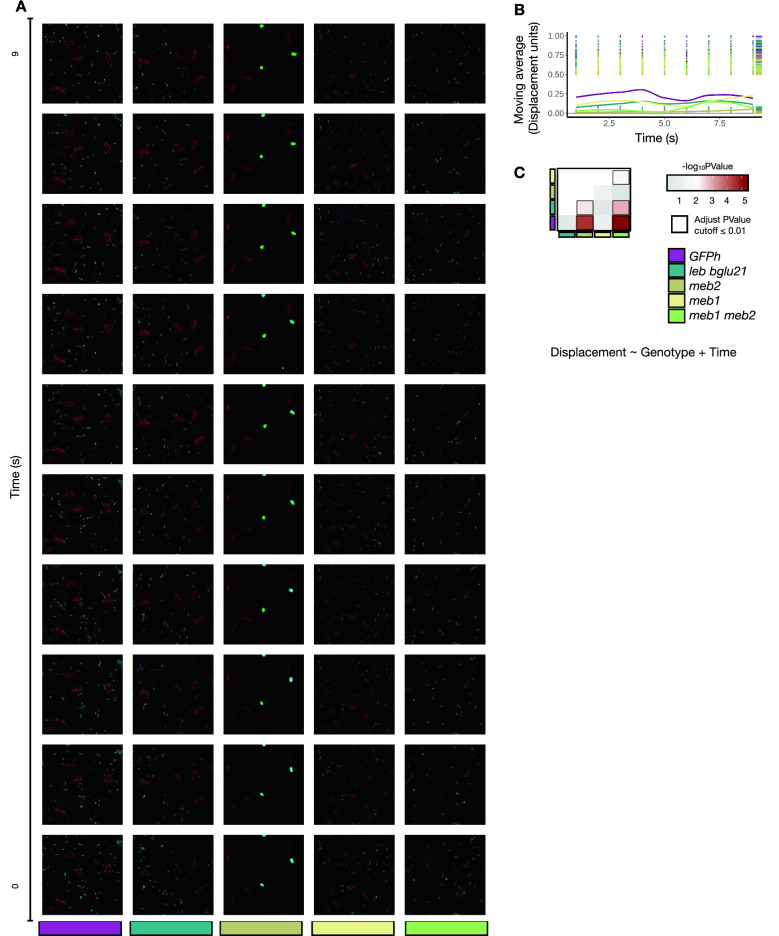
Movement analysis of ER bodies. **A** Sequential images of time-lapse over 10 s. The red channel represents cell-wall, the green channel represents ER body at the respective time (t_n_), the blue channel is dedicated to ER body at the time 0 (t_0_). The separation of the blue channel (t_0_) and the green channel (t_n_) explains ER body displacement in each image. **B** The trend of moving average displacement of ER body features detected within the mutants across time. The mutant genotypes are marked in different colours. The overall distribution is marked on the strip next to the plot. **C** The generalized linear model (GLM) analysis. We used genotype as a fixed factor and time as a covariate. The colour intensity indicates the statistical significance denoted by -log_10_ *p*-value adjusted by Benjamini Hochberg procedure, obtained from the pairwise comparison between the genotypes with the Tukey HSD method. The box indicates the FDR (False Discovery Rate) ≤ 0.01

We used time-lapse image analysis to examine the difference in ER body movement between the wild type and the mutants. We observed ER body movement across time in both wild-type and mutant plants, but noticed that there was a reduction in the ER body movement in the *meb2-1* and *meb1-1 meb2-1* mutants (Fig. 5A and movies in Additional files 9, 10, 11, 12, 13). We calculated the average of the ER body displacements from their initial position and found that the overall ER body movement was highest in the wild type across time (Fig. 5B). A similar trend was observed in the *leb-1 bglu21-1* and *meb1-1* mutants, but not in the *meb2-1* and *meb1-1 meb2-1* mutants. Statistical analysis revealed that movement was reduced in the *meb2-1* and *meb1-1 meb2-1* mutants compared to the wild type, *leb-1 bglu21-1* and *meb1-1* mutants (FDR ≤ 0.01) (Fig. 5C). These findings suggest that the MEB2 protein is involved in ER body movement.

## Discussion

In cell biology the common method for data presentation is showing microscopic images that are only able to provide limited information. It is challenging to distinguish the different morphology of biological objects by image profiling after the calculation of the mathematical parameters, especially for morphologically similar objects like ER bodies. In this paper, by performing statistical data analyses and gathering quantitative data from microscopic images, we successfully extracted more information about the differences between the wild type and mutants of *A. thaliana*. Our analysis clearly shows that the *nai1-1* and *leb-1 bglu21-1* mutants have morphologically distinct ER bodies from the wild type. Additionally, we found that the *meb1-1*, *meb2-1* and *meb1-1 meb2-1* mutants have a distinct profile as well. Our results and methodology are important for studying and distinguishing morphologically similar, yet different, objects in confocal micrographs.

Segmentation of whole microscope images into cells provides detailed information and features at the single-cell level [23]. Considering the potential variety of ER body morphology between the cells, we implemented our method at the level of segmented cells. Mean or average intensity projection and maximum intensity projection of z-stack images are commonly used in studies related to cell organelle. These methods are mostly used to determine the focal plane of the micrographs and sometimes over-represent the elements that are beyond the focus range [31]. Thus, the pixel elements along the z-scores are difficult to distinguish. Recently, a new merging tool, namely *MaxContrastProjection*, was developed that considers the difference across pixels on merging the images and that decreases the noise across z-stack micrographs [21]. In this study we successfully segmented the plant cells with the maximum contrast projection method.

The Haralick features along with the spatial and intensity features provided additional information to investigate the morphological variants of ER bodies. Haralick features provided fourteen descriptors of textural features that come from the co-occurrence matrix, a known statistical approach for texture features. In our analysis we considered 13 features that estimated the range in the lower and higher values as s1 and s2 [22], these features are angular second moment, contrast, correlation, variance, inverse difference moment, the sum of average, the sum of entropy, sum of variance, entropy, the difference in variance, the difference in entropy, and information features of correlation ƒ12 and ƒ13 [32]. In total we estimated 26 Haralick, 6 spatial and 8 intensity features. Subsequent k-means clustering of these features showed representatives that had discrete features within genotypes. The statistical analysis of the features revealed significant differences between the wild type and mutants, not only in spatial parameters, but also in morphological parameters. Indeed, our image- and cell-wise analysis successfully distinguished the morphological diversity of already reported ER body mutants, such as *nai1-1* and *leb-1 bglu21-1* [14, 15]. Nagano et al. reported that the *leb-1 bglu21-1* mutant had a significantly smaller number of ER bodies than the wild type, and that the mean area of an ER body in the *leb-1 bglu21-1* mutant was significantly larger than that in the wild type [14]. Our analysis is consistent with this previous report, as we found an increase of ER body sizes in the *leb-1 bglu21-1* mutant in comparison to the wild type. Furthermore, it was evident that the ER body morphology observed in the *meb1-1, meb2-1* and *meb1-1 meb2-1* mutants differed from the wild type in both the segmented cell and the grouped images. Thus, our analysis provides a robust estimation of morphological diversity of cell compartments like ER-bodies.

Additionally, we introduced position features to measure the ER body movement in time-lapse images. The calculation of the displacement from the location of features gives a better estimate for the ER body movement along time. However, the total displacement of the ER bodies can include their initial position and the z-axis. By using the maximum contrast projection, we were able to retain the pixel information along the z-axis before segmentation of the cells. We measured the total pathway and velocity of ER bodies by considering all the x-, y- and z-axes in the time-lapse images. Additionally, we calculated the cosine distances between the initial and next positions to compute the moving average. The estimated moving average displacement provided a better approximation of the total displacement irrespective of its position across time. Based on these analyses we observed that the movement of the ER bodies are different in the *meb2-1* and *meb1-1 meb2-2* mutants. This suggests that MEB2 may be associated with the movement of the ER bodies.

Despite the difference in PI staining settings implemented during microscopy the morphological diversity of the ER bodies was preserved, and the variation could be objectively distinguished in the CCA. In the integrative analysis we could separate morphologically distinct ER body images according to CCA1 and CCA2 values and cluster them together. The mean of the morphological variation in the segmented image clusters gives a significant resolution of ER body phenotyping in the CCA. Therefore, we could capture the difference between the *leb-1 bglu21-1* mutants and the wild type in the length of their ER bodies. We captured mutants lacking in MEB proteins that showed ER body morphological variation different from the wild type.

It has previously been reported that there are no observable differences in ER body formation between the *meb1-1*, *meb2-1*, *meb1-1 meb2-1* mutants and wild type plants, suggesting that MEB1 and MEB2 are not essential for ER body formation [17]. However, with our methodology of qualitative and quantitative microscopic image analysis, we showed that the morphological parameters in the Haralick features along with the intensity and spatial features significantly distinguished the *meb1-1*, *meb2-1* and *meb1-1 meb2-1* mutants from the wild type. Indeed, with k-mean clustering followed by MDS and CCA analysis, we found that these mutants have irregular ER body phenotypes compared to the wild type. We noticed that ER bodies seem to be sparser in the *meb1-1* and *meb1-1 meb2-1* mutants when compared to the wild type. These findings suggest that MEB1 and MEB2 proteins play a significant role in providing the shape of ER bodies without changing the concentration of the entire proteins PYK10/BGLU23 and NAI2, which are involved in the ER body formation [17]. MEB1 and MEB2 are ER body-specific proteins within the multi-spanning transmembrane regions, which belong to the VIT family [16, 17]. Thus, a deficiency of these proteins may change the membrane organization of ER bodies and further alter the shape of ER bodies. We found that the *meb2-1* mutant reduced ER body movement when compared to the wild type. Therefore, MEB2 may interact with the ER network proteins and motor proteins that are required for ER movement [33].

Nagano et al. distinguished stomata cells using a random forests-based technique on images taken from cotyledon samples to analyse ER body morphology [14]. However, to our knowledge there is no definite classification method that distinguishes ER bodies from ER network over a large dataset from different plant tissues. We report that within a population of segmented cells there is a clear difference between ER bodies and ER network even in the image with different settings. Furthermore, we distinguished ER bodies that are morphologically different among the genotypes. Future studies on ER body morphology and ER movement may take advantage of machine learning and deep learning methods using morphological features extracted from images to train a classification method. However, a vast array of image datasets might be required to classify and distinguish different morphologies of ER bodies with such methods. More than 100 K segmented images and retained morphological properties may be required in training a neural network like Siamese neural network [34] or an additive neural network model in order to accomplish such a task.

In the overall analysis of morphological properties with our method, we have shown that the ER body shape is indeed modulated in the *meb1-1*, *meb2-1* and *meb1-1 meb2-1* mutants. Additionally, we detected variations in ER body movement using simple confocal micrographs. Independent of the recent advances in image processing and image analysis by deep learning, our methodological approach comprehensively characterises different morphologies and movement of cellular compartments that are as small as ER bodies. As of now, there is no neural network optimised for ER body classification across different datasets. The caveat is that deep learning methods require a large dataset to overcome overfitting issues in classifying organelles that are similar in morphology. The methodology presented in this paper describes the difference in morphological features of segmented cell populations depending on their genotype. It will, therefore, help screen mutants that show variation in morphology and dynamics of cellular components under different conditions in an unbiased manner. Additionally, it may also be used in characterising other cellular compartments like chloroplasts, mitochondria, peroxisomes, protein bodies, lipid bodies and starch granules.

## Conclusion

We developed a method to quantitatively analyse confocal microscopy images and obtained different ER body phenotypes. With our methodology we were able to describe morphological diversity of ER bodies that had not been recognised in a previous study. Furthermore, our method enables robust phenotyping of mutants based on cellular and subcellular morphological changes by extracting precise information from complex micrograph images. We found morphological changes of ER bodies in the *meb1-1*, *meb2-1* and *meb1-1 meb2-1* mutants, indicating that ER body membrane proteins MEB1 and MEB2 affect ER body shape. Additionally, we found that the movement of the ER bodies were reduced in the *meb2-1* and *meb1-1 meb2-1* mutants. These finding provide deep insight into the molecular mechanism of ER body formation. Because our analysis provides an estimate of morphological variations and movement patterns, it can be extensively useful for characterising phenotypes of mutants in forward and reverse genetic approaches. Further molecular experiments will reveal the functional association of the factors that show the differences in morphology and movement within the phenotypes.

## Supplementary Information

**Additional file 1.** The parameters used as the default for segmentation of cells.

**Additional file 2.** Detailed schematics of image acquisition, image processing, segmentation and data analysis for the quantification of ER body morphology and dynamics.

**Additional file 3.** Detailed schematic for the cell segmentation describing projection, mask using the global parameter file, the segmented cells and image.

**Additional file 4.** Definition of the parameters used for morphological analysis and dynamics.

**Additional file 5.** Multivariate analysis showing the relationship between the samples, segmented cells in MDS-2 and MDS-3.

**Additional file 6.** Demonstrating the optimisation of *k* using AIC, over a range of *k*-values (3 to 100) chosen on the basis of the number of genotypes (i.e., 3) and 10 sub-clusters within each genotype.

**Additional file 7.** Summary of the constrained and unconstrained ordination conducted with Pearson correlation as a distance matrix on a normalised feature matrix.

**Additional file 8.** The image feature variation within wild type and mutants.

**Additional file 9.** Movies showing the differences in the ER body dynamics of wild type (GFP-h).

**Additional file 10.** Movies showing the differences in the ER body dynamics of *leb-1 bglu21-1* mutant.

**Additional file 11.** Movies showing the differences in the ER body dynamics of *meb2-1* mutant.

**Additional file 12.** Movies showing the differences in the ER body dynamics of *meb1-1* mutant.

**Additional file 13.** Movies showing the differences in the ER body dynamics of *meb1-1 meb2-1* mutant.

## Data Availability

The scripts used can be found in the GitHub repository, images can be found in the Bioimage database. Scripts used for pre-processing and analysis along with supplementary data can be found in http://www.github.com/arpankbasak/ER_DynaMo. The analysis environment erb_dynamo with all necessary dependencies have been uploaded to https://anaconda.org/arpankbasak/erb_dynamo/files. The plant materials are available from the Arabidopsis Biological Resource Center (ABRC) and Nottingham Arabidopsis Stock Center (NASC).

## Abbreviations

BGLU: β-Glucosidase
CCA: Constrained canonical analysis
ER: Endoplasmic reticulum
FDA: Flexible discriminant analysis
MDA: Mixture discriminant analysis
MDS: Multidimensional scaling
MEB: Membrane of ER body
PI: Propidium iodide
VIT: Vacuolar iron transporter
