# Supplementary material for: Texture feature extraction from microscope images enables a robust estimation of ER body phenotype in Arabidopsis
Source: Plant Methods. 2021 Oct 26;17:109. doi: 10.1186/s13007-021-00810-w (PMC8549183; doi:10.1186/s13007-021-00810-w)
Supplement: Supplementary file 1 — Additional file 1. The parameters used as the default for segmentation of cells. [file 13007_2021_810_MOESM1_ESM.pdf]

Additional file 1. The parameters used as the default for segmentation of cells

Mode	Parameter	Default Values	Description
segregate	time_points	0	Number of time points in 4D based analysis [0: Not a time-series experien
	dimension	1024	The resolution of the image
	channel_red	2	Input channel number dedicated to red [Cell]
	channel_green	1	Input channel number dedicated to green [feature]
	channel_blue	3	Input channel number dedicated to blue
	cell_channel	1	Output channel for the cell border [1: Red]
	cell_window_x	3	Matrix for cell operation
	cell_window_y	3	
	cell_brush_shape	box	Type of brush used for cell operation
	cell_smoothing	5	Smoothing parameter for cell
	feature_channel	2	Output channel for the feature [2: Green]
	feature_window_x	1	Matrix for feature operation
	feature_window_y	1	
	feature_brush_shape	box	Type of brush used for feature operation
	feature_smoothing	5	Smoothing parameter for feature
segmentation	kernal_brush_size	51	Kernel size set for cell-like objects
	kernal_brush_shape	gaussian	Kernel brush shape
	kernal_brush_sigma	1	Kernel brush sigma
	feature_otsu_threshold	0	Otsu threshold for feature (optional)
	feature_q_threshold	0.96	Quantile threshold for features (optional)
	feature_opening_w	10	Opening operation for features (w: width, h: height)
	feature_opening_h	10	
	feature_opening_offset	0.0001	Offset parameter for opening operation
	feature_opening_brush_width	3	Opening brush width
	feature_propagate_lambda		Propagation of features with tolerance lambda (optional)
	feature_opening_brush_shape	disc	Brush shape for opening parameter
	void_brush_size	3	Brush size for marking the void
	void_brush_shape	disc	Shape of the brush used for marking the void
	void_brush_q50	0.5	Median of the void
	void_otsu_threshold	0	Otsu threshold for void (optional)
	void_opening_w	20	Marking the void for opening operation
	void_opening_h	20	
	void_opening_offset	0.000001	Offset parameter for opening operation
	void_opening_brush_width	3	Opening brush width
	void_opening_brush_shape	disc	Brush shape for opening parameter
	void_propagate_lambda		Propagation of void with tolerance lambda (optional)
	void_brush_qrange	0.65	Quantile range for void detection
	cell_brush_size	3	Brush size for marking the cells
	cell_brush_shape	disc	Shape of the brush used for marking the cells
	cell_brush_qlimit	0.95	Quantile threshold for cells
	cell_hull_offset	0.000001	Hull offset used for segmenting cells
	cell_hull_h	20	Matrix used for Hull operation
	cell_hull_w	20	
	cell_otsu_threshold	0	Otsu threshold for feature (optional)
	cell_opening_qlimit	0.99	Threshold limit for cell opening
	cell_opening_brush	0.01	Brush for opening operation
	cell_opening_offset	0.01	Offset for opening operation
	cell_opening_brush_width	3	Cell opening brush width
	cell_opening_brush_shape	disc	Shape of the brush used for cell opening operation
	cell_propagate_lambda	1E-04	Propagation parameter
	cell_rarefry_surface_area	1000	Minimum range of surface area for a cell*
	feature_op_otsu_threshold	0	Otsu threshold for feature within segmented cells (optional)
	feature_op_threshold_w	10	Matrix for detecting the features within the segmented cells
	feature_op_threshold_h	10	
	feature_op_threshold_offset	0.0001	Offset threshold
	feature_op_brush_size	3	Feature brush size
	feature_op_brush_shape	disc	Feature brush shape
	feature_op_brush_w	20	Feature brush
	feature_op_brush_h	20	
	feature_op_brush_offset	0.005	Feature offset
cores	mc.cores	12	Number of dedicated CPU cores
