# Supplementary material for: Texture feature extraction from microscope images enables a robust estimation of ER body phenotype in Arabidopsis
Source: Plant Methods. 2021 Oct 26;17:109. doi: 10.1186/s13007-021-00810-w (PMC8549183; doi:10.1186/s13007-021-00810-w)

Additional file 2. Detailed schematics of image acquisition, image processing, segmentation and data analysis for the quantification of ER body morphology and dynamics

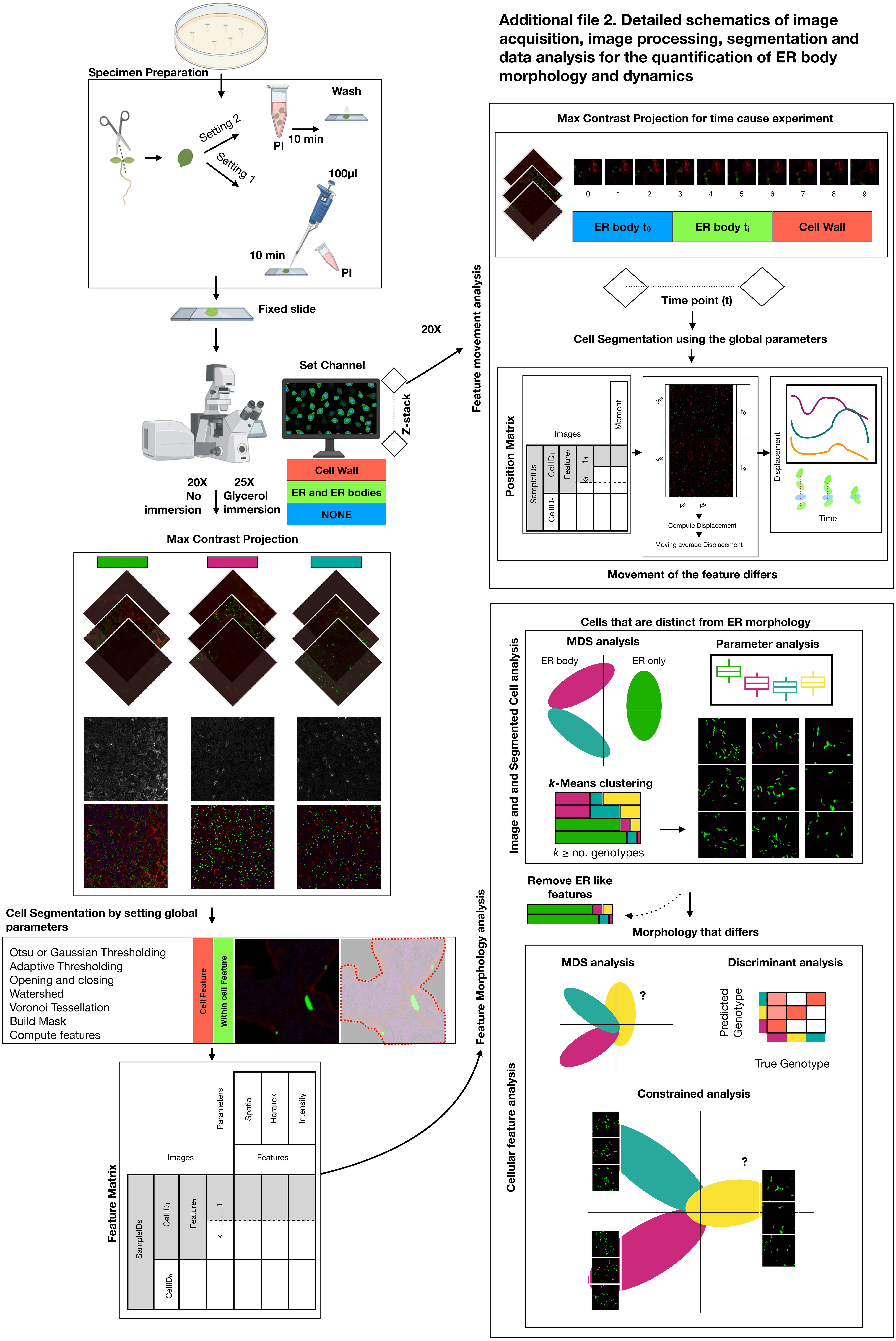

Supplement: Supplementary file 2 — Additional file 2. Detailed schematics of image acquisition, image processing, segmentation and data analysis for the quantification of ER body morphology and dynamics. [file 13007_2021_810_MOESM2_ESM.pdf]
