# Supplementary material for: Texture feature extraction from microscope images enables a robust estimation of ER body phenotype in Arabidopsis
Source: Plant Methods. 2021 Oct 26;17:109. doi: 10.1186/s13007-021-00810-w (PMC8549183; doi:10.1186/s13007-021-00810-w)
Supplement: Supplementary file 3 — Additional file 3. Detailed schematic for the cell segmentation describing projection, mask using the global parameter file, the segmented cells and image. [file 13007_2021_810_MOESM3_ESM.pdf]

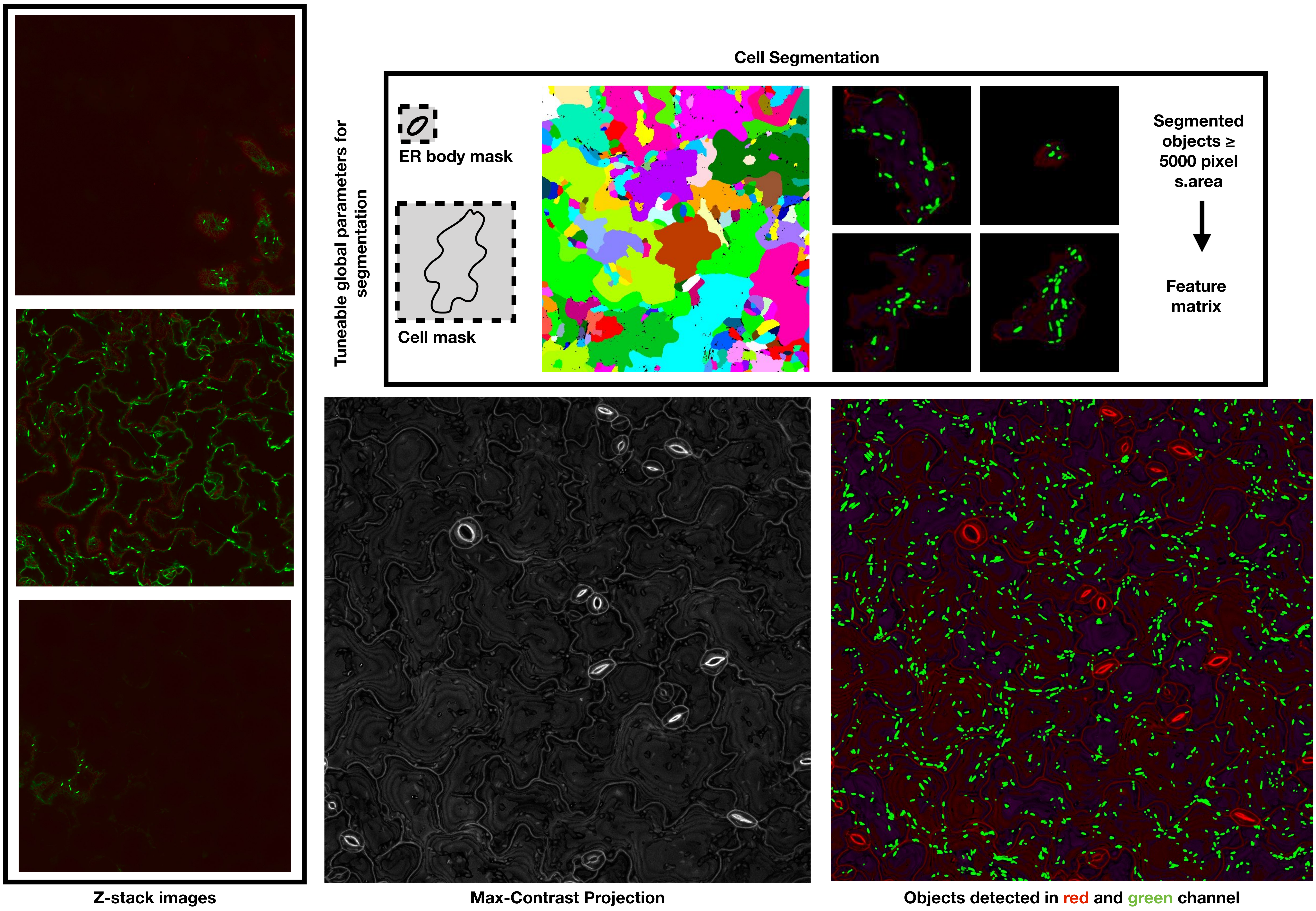

Additional file 3. Detailed schematic for the cell segmentation describing projection, mask using the global parameter file, the segmented cells and image
