# Supplementary material for: Texture feature extraction from microscope images enables a robust estimation of ER body phenotype in Arabidopsis
Source: Plant Methods. 2021 Oct 26;17:109. doi: 10.1186/s13007-021-00810-w (PMC8549183; doi:10.1186/s13007-021-00810-w)
Supplement: Supplementary file 4 — Additional file 4. Definition of the parameters used for morphological analysis and dynamics. [file 13007_2021_810_MOESM4_ESM.pdf]

Additional file 4. Definition of the parameters used for morphological analysis and dynamics

Types of features	Short name	Definition	Description
Intensity			
	b.mean	Mean intensity	Fluorescence intensity features provide independent statistics of pixel distribution of the objects detected in the images
	b.sd	Standard deviation of intensity	
	b.mad	Mean absolute deviation of intensity	
	b.q001	1% Quantile of intensity	
	b.q005	5% Quantile of intensity	
	b.q05	50% Quantile of intensity	
	b.q095	95% Quantile of intensity	
	b.q099	99% Quantile of intensity	
Haralick (Miyamoto 2008)			
	h.asm	Angular second moment	The Haralick features in an image is computed from statical properties of the GLCM. These set of features provide association of the detected objects with neighbouring pixels. Thereby providing estimates morphological differences within the detected objects
	h.con	Contrast	
	h.cor	Correlation	
	h.var	Variance	
	h.idm	Inverse difference moment	
	h.sav	Sum of average	
	h.sva	Sum of variance	
	h.sen	Sum of entropy	
	h.ent	Enropy	
	h.dva	Difference in variance	
	h.den	Difference in entropy	
	h.f12	Measure of correlation coefficient	
	h.f13		
Spatial and shape			
	s.area	Surface area	The shape and spatial properties provide descriptive statistics of the structure of the object detected
	s.perimeter	Perimeter	
	s.radius.mean	Mean radius of the object	
	s.radius.sd	Standard deviation of the mean radius	
	s.radius.max	Maximum radius	
	s.radius.min	Minimum radius	
Position (Used for quantification of movement)			
	m.cx	Centre of mass in x-axis	The centre of mass detects the exact location of the object (x- and y-axis) in the image. This information is further used to calculate the total distance covered by the object in a time-series experiment
	m.cy	Centre of mass in y-axis	
			All measures are in pixels
