# Supplementary material for: Texture feature extraction from microscope images enables a robust estimation of ER body phenotype in Arabidopsis
Source: Plant Methods. 2021 Oct 26;17:109. doi: 10.1186/s13007-021-00810-w (PMC8549183; doi:10.1186/s13007-021-00810-w)
Supplement: Supplementary file 5 — Additional file 5. Multivariate analysis showing the relationship between the samples, segmented cells in MDS-2 and MDS-3. [file 13007_2021_810_MOESM5_ESM.pdf]

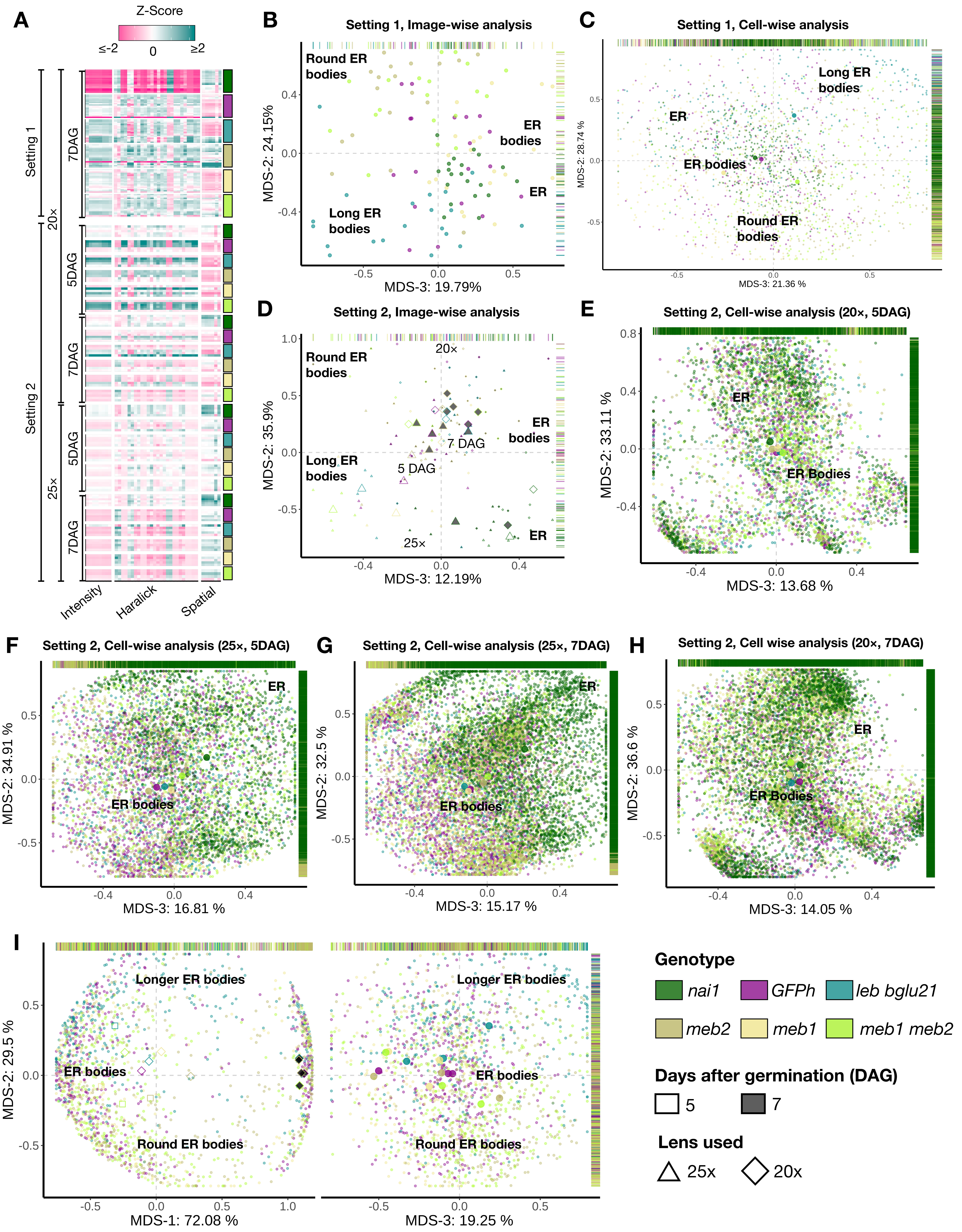

Additional file 5. Multivariate analysis showing the relationship between the samples, segmented cells in MDS-2 and MDS-3

A The heat map represents the z-score of the 40 features (x-axis) and images (y-axis) across two plant age (5 and 7 days after germination (DAG)), two PI staining settings (Setting 1 and Setting 2) and two objective lens (20× and 25×). **B-H** MDS analysis was performed on the z-score of the morphological parameters in the independent dataset. The MDS3 and MDS2 is represent in x-axis and y-axis, respectively, showing the maximum variance explained in the 3rd and 2nd co-ordinates. The MDS analysis represents maximum variation in the genotype from the image dataset using setting 1 (B-C) or setting 2 (D-H). The colours represent the genotype. The MDS analysis for cell-wise images is separated into four scatterplots depend on the objective lens and plant age (E-H). **I** The MDS analysis of the segmented cell images containing ER bodies.
