# Supplementary material for: Texture feature extraction from microscope images enables a robust estimation of ER body phenotype in Arabidopsis
Source: Plant Methods. 2021 Oct 26;17:109. doi: 10.1186/s13007-021-00810-w (PMC8549183; doi:10.1186/s13007-021-00810-w)
Supplement: Supplementary file 6 — Additional file 6. Demonstrating the optimisation of k using AIC, over a range of k-values (3 to 100) chosen on the basis of the number of genotypes (i.e., 3) and 10 sub-clusters within each genotype. [file 13007_2021_810_MOESM6_ESM.pdf]

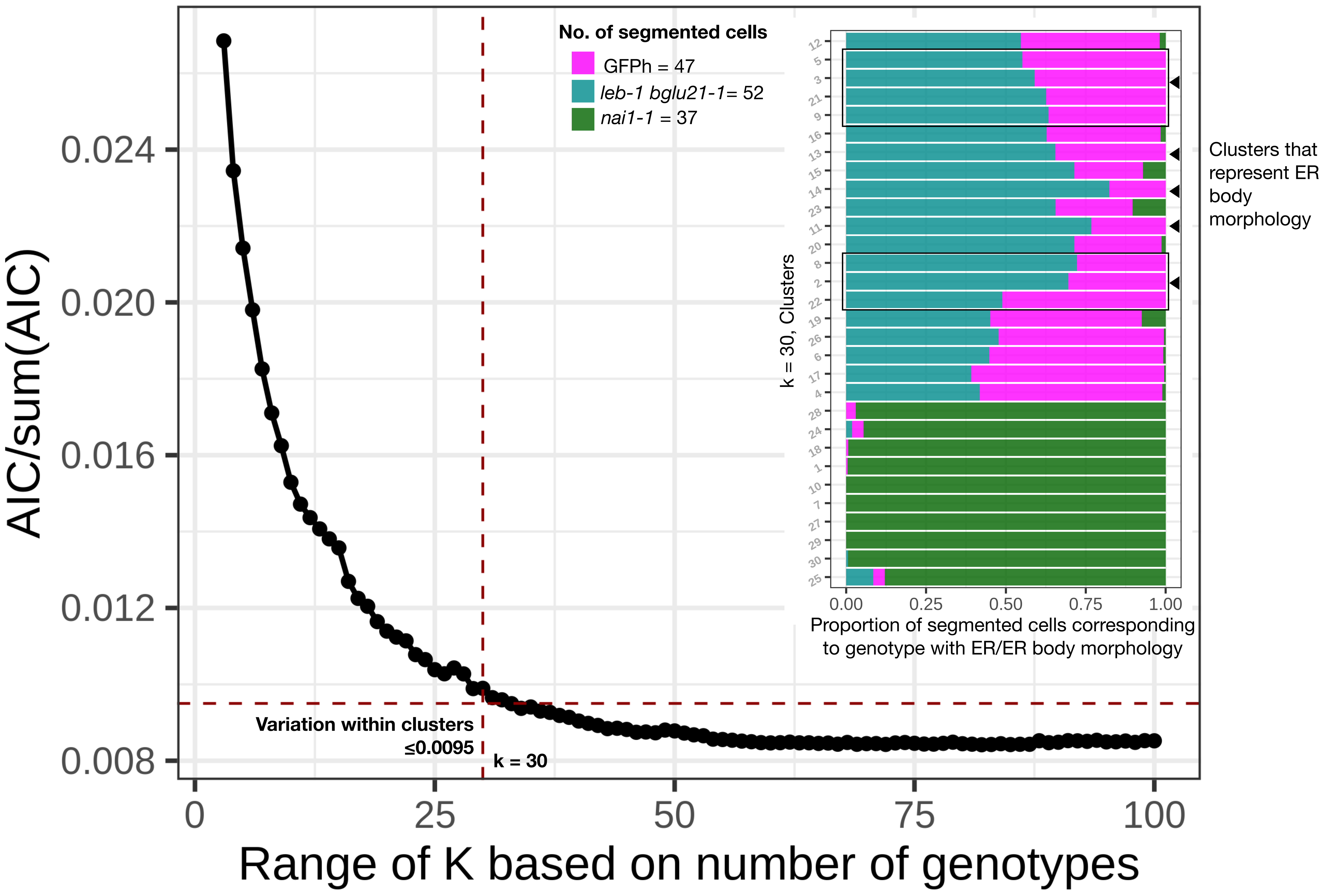

Additional file 6: Demonstrating the optimisation of *k* using AIC, over a range of *k*-values (3 to 100) chosen on the basis of the number of genotypes (i.e., 3) and 10 sub-clusters within each genotype
The demonstration is performed on 3 randomly selected images from GFPh, *leb1-1* and *nai1-1* genotypes.
