# Supplementary material for: Texture feature extraction from microscope images enables a robust estimation of ER body phenotype in Arabidopsis
Source: Plant Methods. 2021 Oct 26;17:109. doi: 10.1186/s13007-021-00810-w (PMC8549183; doi:10.1186/s13007-021-00810-w)
Supplement: Supplementary file 7 — Additional file 7. Summary of the constrained and unconstrained ordination conducted with Pearson correlation as a distance matrix on a normalised feature matrix. [file 13007_2021_810_MOESM7_ESM.pdf]

Additional file 7. Summary of the constrained and unconstrained ordination conducted with Pearson correlation as a distance matrix on a normalised feature matrix

		Inertia	Propotion
**Image wise analysis**	**Total**	77.678	1
	**Constrianed**	5.8763	0.07565
	**Unconstrained**	26.0686	0.33559
**Segmented cell wise analysis**	**Total**	14512.0392	1
	**Constrianed**	199.4591	0.01374
	**Unconstrained**	11253.6069	0.77546
