# Supplementary material for: Texture feature extraction from microscope images enables a robust estimation of ER body phenotype in Arabidopsis
Source: Plant Methods. 2021 Oct 26;17:109. doi: 10.1186/s13007-021-00810-w (PMC8549183; doi:10.1186/s13007-021-00810-w)
Supplement: Supplementary file 8 — Additional file 8. The image feature variation within wild type and mutants. [file 13007_2021_810_MOESM8_ESM.pdf]

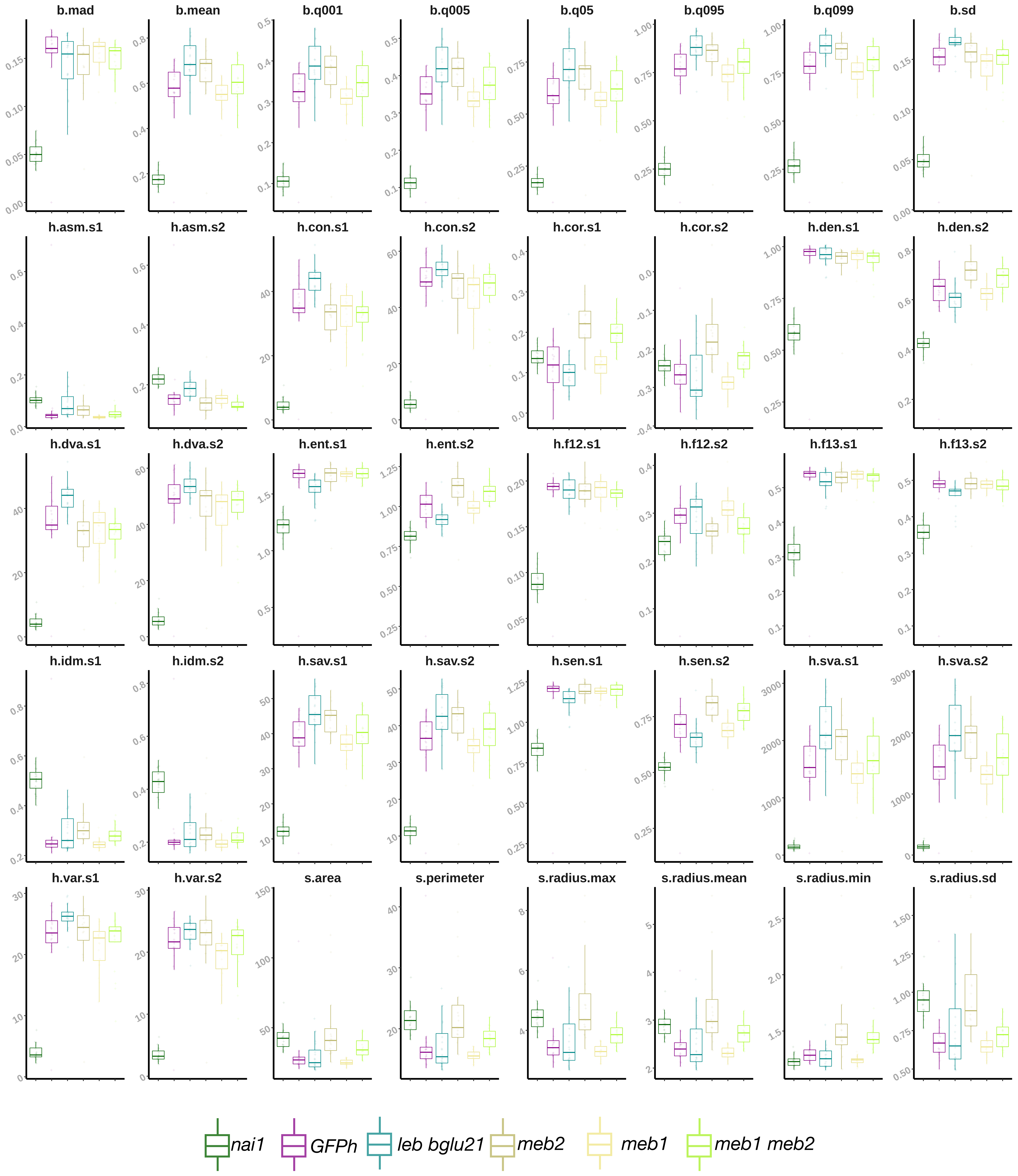

Additional file 8. The image feature variation within wild type and mutants

The box plot represents the variation within the samples estimated from the experiment conducted using PI staining setting 1.
